# Measurement, data analysis and modeling of electromagnetic wave propagation gain in a typical vegetation environment

**DOI:** 10.1371/journal.pone.0280035

**Published:** 2023-01-12

**Authors:** Chaoyi Zhang, Zhangchao Ma, Jianquan Wang, Yan Yao, Xiangna Han, Xiang He

**Affiliations:** 1 Automation and Electronic Engineering College, University of Science and Technology Beijing, Beijing, China; 2 Automation College, Beijing University of Posts and Telecommunications, Beijing, China; 3 Institute of Cultural Heritage and History of Science & Technology, University of Science and Technology Beijing, Beijing, China; Edinburgh Napier University, UNITED KINGDOM

## Abstract

This paper takes the specific environment covered by vegetation as the research object, carries out modeling and analysis, takes the large-scale fading model of wireless channel as the basis of data processing, researches the transmission law of electromagnetic wave in a typical vegetation environment, which can be divided into four situations. The signal attenuation in each case is theoretically derived and numerically simulated. From the view point of supporting vegetation environment channel, the large-scale channel measurement system is built to meet the actual needs, such as bandwidth, frequency, vegetation coverage, etc. the final vegetation environment channel model under the large-scale fading model is obtained. The results show that the path gain of four scenarios respectively are 81.3 dB, 36.5 dB, 1.6 dB, 1.5 dB, the value of path gain index is within the range of 2~3.5, four scenarios shadow fading standard deviation values are 7.1, 4.8, 10.1, 9.2, reflects the change of received power at the point caused by random factors such as reflection, absorption and scattering. In addition, the proposed channel model improves the gain about 15% compared with the tradition SUI model within vegetation coverage scene. The design process of the proposed model is carried out in the order of "studied the existing foundation → analyzed the existing problems → proposed the optimization scheme → simulation and verification results → actual measurement system". The advantage of paper’s method is that, when the signal frequency, transceiver distance, antenna height and vegetation environment characteristic parameters are given, the statistical analysis results of wireless channel data are obtained. The purpose of the proposed work establishes a signal propagation prediction model under the vegetation environment, realizes a theoretical basis for channel simulation, and provides the basis of anti-fading technologies.

## Introduction

Vegetation environments have a great impact on the propagation of radio waves. The gain of radio waves in a vegetation is much greater than in the atmosphere [1–3]. Wireless signals are affected by the topography, vegetation, weather and other factors during the propagation process: reflection, scattering, absorption and other phenomena may cause signal attenuation [4–6]. The electromagnetic signal transmission model has always been the focus of scholars in the field of electromagnetics. Such models currently include the Okumura-Hata [7] model, the Egli model [8], the ITU-R model [9] and the COST231 model [10] and are simple in form. The environment (city, suburb, ocean, etc.) has a single spatial distribution and regular shapes of objects, but these theoretical models produce errors when predicting radio wave transmissions in complex vegetation environments; thus, this channel needs to be analysed from the perspective of electromagnetic signal transmission.

At present, primarily theoretical analysis methods and field measurement methods have been used for research into the propagation of radio waves in vegetation. They have different emphases. Theoretical analysis methods go deep into the propagation theory of the physical processes of electromagnetic wave propagation in the media, mainly including the reflection, scattering and absorption of the propagation process [11–13]. These methods need to use numerical analysis approaches to establish a complex mathematical model. Measurement methods use experiments and derivations, which have strong limitations for different environments [14,15]. The theoretical analysis method mainly consists of two kinds of models. The first kind of model describes vegetation as a layered medium, with each layer a uniform or uneven lossy material. For example, Giarola, Calvaceante and Tamir [16], et al. divide the canopy into three layers of lossy medium, with each layer described as a unique dielectric to ensure that it is the same isotropic material considering the electromagnetic waves passing through each layer [17,18]. After each layer boundary, there are more detailed 4-layer models, which distinguish the canopy, branch and trunk, and more accurate modelling can be performed [19–21]. The second model treats vegetation as a whole without any separation. This whole body is composed of mixtures of different materials. Matzler and Lagrone regard the canopy as a mixture of branches, leaves and air. Each material is represented by a simple geometric model that has electromagnetic parameters such as a complex conductivity. Finally, the canopy model is established according to the statistical distribution of the spatial directions of each element. The field measurement method is simple in calculation and wide in generality. It has strong practicability in wireless coverage predictions under specific environments. Weissberger first proposed the idea of calculating the propagation gain of radio waves in vegetation by taking the propagation distance and frequency as parameters [22,23]. In addition, Brazil Costa Dias M. H. et al. [24], American Tian Y. D., et al. [25] tested the propagation of electric waves in a tropical rainforest and finally determined the gain formula of the tropical rainforest. Chen and Kuo in China also determined models for propagation losses in vegetation at a horizontal and vertical polarization through experimental analysis, and gave different parameter selections [26].

Based on the Tamir model, this paper studies the transmission laws of electromagnetic waves in typical vegetation environments. The main mathematical modeling process of Tamir model, is to research the attenuation law of the signal propagation in forest. The Tamir model considers the dielectric constant of vegetation canopy, the diffraction, refraction and reflection of electromagnetic waves in the forest, introduces the concept of “side wave” to establish a complete propagation gain model of electromagnetic waves in the forest. The applicable scenarios of other channel models [7–10] are mostly used in urban, rural and other hot spot areas, which are quite different with ‘forest’ environment. Therefore, these channel models [7–10] are not used in this paper.

The theoretical derivation and numerical simulation of signal attenuation is carried out, the results show that the attenuation model of electromagnetic wave signals proposed in this paper is real and reliable, which can provide a basis for wireless channel simulations in vegetation environments and theoretical support for various anti fading technologies.

## Path gain prediction of equivalent model under vegetation cover

The Tamir model [16] is equivalent to a layered lossy medium divided into three layers: ground-vegetation-air. Each layer has different electrical parameters. The idea of stratification is used to solve the problem. Another important factor of radio wave propagation in vegetation is the positional relationship between the transmitting and receiving antennas relative to the vegetation. When the receiving antenna is placed in the above different positions, there are different propagation mechanisms, we will discuss the transmission losses when there are trees on the transmission path between the transmitter and receiver. 1. It is a vegetation area. 2. It is a vegetation path where the transmitter is located in the free space. 3. It is a wave propagating in the open space area in the vegetation. 4. It is a combination of trees and buildings in line with the radio propagation.

In these four scenarios, the frequency range of electromagnetic wave is 200MHz~2.6GHz, which covers the range from low to high frequency, consistent with the frequency range of mobile communication systems. The environment temperature is 25°C and the humidity is 42%. As the artificial forest is selected as the research object, the dielectric constant of vegetation is between 6~10 in the transmission frequency range. These pre-conditions are suitable for the propagation of electromagnetic waves in the vegetation covered environment.

### A. Transmitter and receiver are located in the vegetation covered area

In practical applications when building the propagation prediction model, the most commonly considered situation is that the receiving antenna is located in the vegetation. Because the electromagnetic waves mainly consider the lateral waves when they propagates in the vegetation for a long distance, we next will primarily consider these lateral waves.

**Fig 1:**
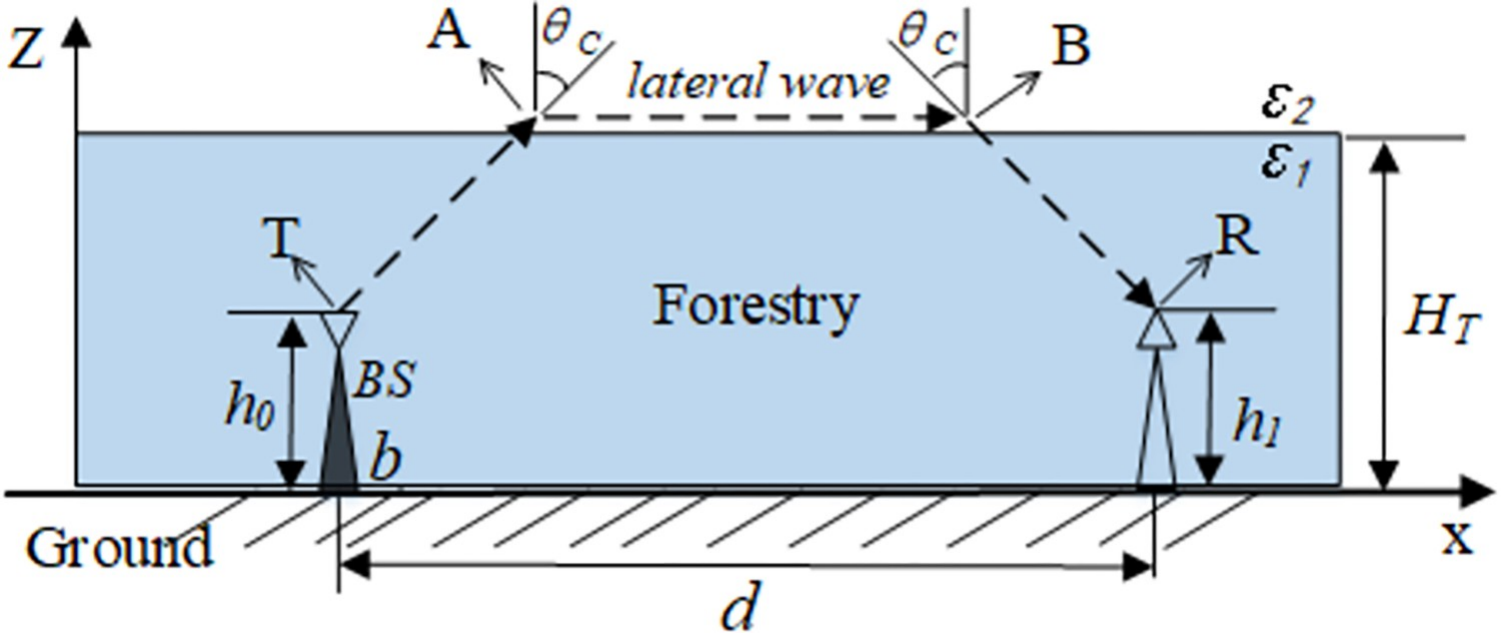
Vegetation signal propagation through lateral waves.

As shown in Fig 1, microwave transmission is N-LOS (non line of sight), the shadow area is the equivalent vegetation coverage, the ground is the x-axis, the air dielectric constant is *ε*_2_, the spatial height of vegetation coverage is z-axis, the vegetation dielectric constant is *ε*_1_, assuming the height of vegetation canopy is *H*_*T*_, the signal transmitter is located at *x* = *b*, the height of transmitter is *h*_0_, and the signal receiver is located at the distance *d* from the transmitter, the height of receiver is *h*_1_, points A, B are the places where the electromagnetic wave shoots out and into the vegetation canopy. The dotted line along the vegetation canopy in the air is the side wave. The exit and incidence angle of the electromagnetic wave at point A and B are both *θ*_*C*_, the dotted line and arrow direction in Fig 1 are the paths of electromagnetic waves from the transmitter to receiver. The lateral wave plays a major role. It can be derived in the range of *x*>*b*, 0<*z*<*H*_*T*_ for the whole region; then, when the distance between the transmitting and receiving antenna is *x* = |*d*|, the field strength can be obtained as follows:

$${\tilde{E}}_{1}=\frac{60Il}{(n^{2}-1)x^{2}}e^{j[k|x|+k_{l}(2H_{T}-h_{1}-h_{0})]}F(90^{o},h_{0})$$ (1)

where *k* = 2*π*/*λ* = 2*πf*(*u*_0_*ε*_0_)^1/2^ is the plane propagation factor in the air, *u*_0_ is the permeability in the air, *f* is the signal frequency of the transmitting antenna, *k*_*L*_ = *k*(*n*^2^−1)^1/2^, *I* is the current on the dipole, *l* is the length of the dipole, and the ground plane reflection coefficient is as follows:

$$F(\theta_{C},h_{1})=\frac{1+B(\theta_{C},h_{1})}{1-B(\theta_{C},H_{T})}$$ (2)

The reflection of the ground plane will return part of the energy to the vegetation air interface and will form the side waves again. Although it is not the main wave, it will still affect the amplitude of the side wave. For larger values of *h*_0_ and *H*_*T*_, the side wave formed by the reflection from the ground can be ignored. In this case, *F*(90°, *h*_1_) is close to 1, and:

$$B(\theta_{C},h_{1})=d_{⊥,//}(\theta_{C})e^{j2k_{L}h_{1}}$$ (3)

$$B(\theta_{C},H_{T})=d_{⊥,//}(\theta_{C})e^{j2k_{L}H_{T}}$$ (4)

where *θ*_*C*_ is the incident angle and *d*_//_(*θ*_*C*_) and *d*_⊥_(*θ*_*C*_) are the reflection coefficients of horizontal and vertical polarization waves at the vegetation-floor ground interface, respectively.

The final expression of the electromagnetic field of the Tamir model in region 1 is shown in Eq [27]. In the above model, the path of side waves is T-A-B-R, where the length of each segment is: *TA* = (*H*_*T*_−*h*_0_)*secθ*_*C*_, $AB=x-[(H_{T}-h_{0})tan\theta_{C}+(H_{T}-h_{1})tan\theta_{C}]$, and *BR* = (*H*_*T*_−*h*_1_)*secθ*_*C*_. From Fig 7, the total electrical length is:

$$kn(|TA|+|BR|)+k|AB|=k(ns\cdot sec\theta_{C}+x-s\cdot tan\theta_{C})=k(|x|-\sqrt{n^{2}-1}s)=k|x|+k_{L}s$$ (5)

where *s* = 2*H*_*T*_−*h*_1_−*h*_0_, $\theta_{c}=arcsin(1/\sqrt{\varepsilon_{r}})$ is the critical angle for generating side waves. Long-distance wireless transmissions are completed by the side waves parallel to the canopy. The side waves are generated by a beam that reaches the canopy. The transmitting angle of the beam is its critical angle, so the critical refraction occurs at the canopy. In the case of neglecting the ground reflections and assuming that the horizontal distance *d* between the sender and the receiver is far greater than the height of the vegetation, the path gain can be obtained as follows:

PG=(1/|χ|)2(λ/2πd)4exp[2S⋅c⋅Im(χ)] (6)

The units of path gain *L* are dB, i.e., L = 10 *logPG*, where *S* is calculated as follows:

$$S=(H_{T}-h_{0})+(H_{T}-h_{1})$$ (7)

### B. Prediction gain of vegetation path with transmitter in free space

**Fig 2:**
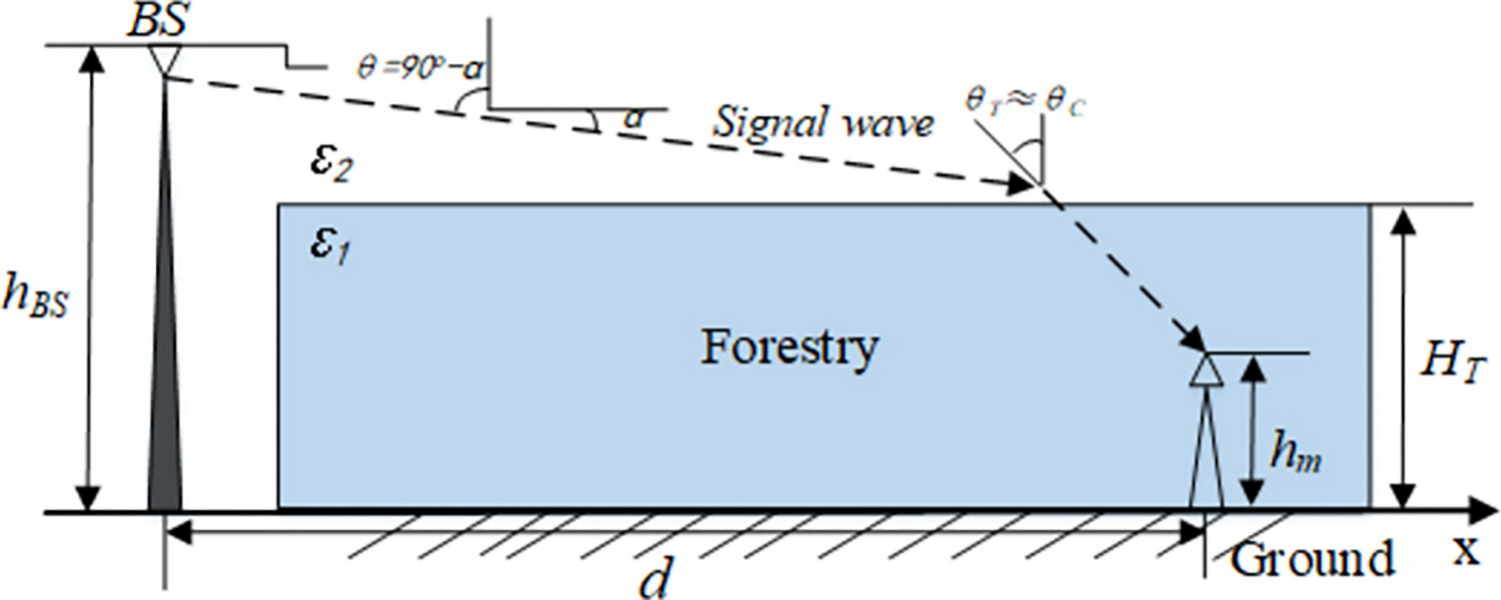
Prediction gain of vegetation paths with the base station in free space.

Fig 2 shows the propagation of base stations taller than the trees (*h*_*BS*_>*H*_*T*_), microwave transmission is a combination of LOS (line of sight) and N-LOS. When *d* is large, the total path gain can be obtained from the free space path gain, tree canopy path gain and attenuation from the tree canopy to the user. The physical transmission process and causes of electromagnetic wave in this scene are as follows: the radio frequency transmitting part of BS is located above the vegetation cover, as shown in Fig 2, the signal is sent from BS to RS (inside forest). According to Tamir model, the whole path of electromagnetic wave signal is the direct path from BS to the vegetation canopy in free space, and the vegetation canopy to RS at the signal receiving end. Therefore, when calculating the path gain, it is necessary to calculate the direct path gain from BS to vegetation canopy in free space, and the gain from vegetation canopy to RS. the total path gain is composed of the signal attenuation of these two parts. The calculation and derivation of each part have been introduced in the above Tamir model and will not be repeated here. According to Tamir model and electromagnetic wave derivation in free space, the total path gain can be calculated as follows:

PG=(λ/4πd)2|T|2exp[2(HT−hm)cIm(ε1−sin2θ)] (8)

Eq (8) is obtained according to Tamir model and attenuation law of electromagnetic wave transmission in free space, where *θ* is the incident angle measured in the vertical direction, (*λ*/4*πd*)^2^ represents the effective area of the receiver antenna, *λ* represents the wavelength, *d* is the propagation distance, *T* representspropagation coefficient, *H*_*T*_−*h*_*m*_ is the distance from the vegetation canopy to the receiver, i.e., the distance from the free space to receiver in vegetation coverage, *c* is the transmission rate of electromagnetic wave, Im(ε1−sin2θ) represents the imaginary part of the electromagnetic wave attenuation, where it represents the change of signal phase (incident angle), so, exp[2(HT−hm)cIm(ε1−sin2θ)] represents the phase change of electromagnetic wave attenuation in vegetation coverage. Eq (8) is the path gain of the received signal on the path (dotted line in Fig 2).

When *d* is large, Eq (8) is quite close to the true value. Using Snell’s law, when *α* is small in Fig 2, there are:

cosθ=sinα≪1 (9A)

$$cos\theta_{T}=\sqrt{1-\frac{1}{\varepsilon_{1}}sin^{2}\theta }=\sqrt{1-\frac{1}{\varepsilon_{1}}cos^{2}\alpha }\approx \sqrt{1-\frac{1}{\varepsilon_{1}}}$$ (9B)

Using Eq (9) and

$$T_{E}\equiv \frac{V^{T}}{V^{I}}=1+\Gamma_{E}=\frac{2cos\theta }{cos\theta +\sqrt{\varepsilon_{1}}cos\theta_{T}}$$ (10A)

$$T_{H}\equiv \frac{I^{T}}{I^{I}}=1+\Gamma_{H}=\frac{2\sqrt{\varepsilon_{1}}cos\theta }{\sqrt{\varepsilon_{1}}cos\theta +cos\theta_{T}}$$ (10B)

The transmission coefficients of TE and TM polarization waves are:

$$T_{E}=\frac{2cos\theta }{cos\theta +\sqrt{\varepsilon_{1}}cos\theta_{T}}=\frac{2sin\alpha }{sin\alpha +\sqrt{\varepsilon_{1}-1}}$$ (11A)

$$T_{H}=\frac{2\sqrt{\varepsilon_{1}}cos\theta }{\sqrt{\varepsilon_{1}}cos\theta +cos\theta_{T}}=\frac{2\varepsilon_{1}sin\alpha }{\varepsilon_{1}sin\alpha +\sqrt{\varepsilon_{1}-1}}$$ (11B)

Because |*ε*_1_-1|>0.01, $\sqrt{\varepsilon_{1}-1}$>0.1. Therefore, *sinα* can be ignored in Eq (11). In addition, due to 0.1>|*ε*_1_-1|, it can be considered that *ε*_1_*sinα*≈*sinα*. Under the above conditions, the propagation coefficient can be written as:

$$T=\frac{2sin\alpha }{\sqrt{\varepsilon_{1}-1}}\approx \frac{2(h_{BS}-H_{T})}{d\sqrt{\chi }}$$ (12)

Using Eq (12), set *sin*^2^*θ*≈1 in Eq (8), and the path gain can be written as:

PG=(λ4π)24d4(hBS−HT)2|χ|exp[2(HT−hm)⋅c⋅Im(χ)] (13)

### C. Radio wave propagation in open space vegetation area

**Fig 3:**
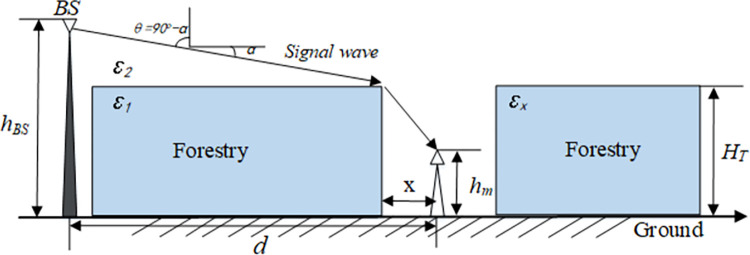
Radio wave propagation in open space areas of a vegetation.

The path gain of radio wave propagation model in the open space area of a vegetation is shown in Fig 3, where the edge of vegetation is the diffraction area.

This model is derived from the measurement results of LaGrone [28], microwave transmission is LOS. The specific physical process is as follows: the edge of the vegetation is equivalent to the edge diffraction angle which can produce horizontal polarization wave at 82MHz~2.95GHz. For users close to the vegetation, the height of the equivalent edge is smaller than the actual height of the tree at a low frequency, which indicates that there is penetration in the leaves. However, when the user is far away from the vegetation, the height of the equivalent edge in the UHF band is close to the actual height of the trees. If there is a path through the vegetation, the attenuation on the path at 457 MHz and 914 MHz varies with the seasons, and the difference can reach several dB, indicating that the equivalent height depends on the type of attenuation. In the following analysis, we assume that the height of an equivalent blade is equal to the height of the trees.

The field strength of the canopy is the incident field strength plus the reflected field strength, which is (1+Γ) = *T* times the incident field strength. The electromagnetic field reaches the ground by diffraction. If *d* is much larger than the distance *x* from the edge of the vegetation to the user and the ground reflection is ignored, the path gain from the base station to the user is:

$$PG={\left(\frac{\lambda }{4\pi d}\right)}^{2}|T{|}^{2}\frac{|D{|}^{2}}{\rho }$$ (14)

where *D* is the edge diffraction coefficient (Fresnel zone), $\rho =\sqrt{x^{2}+{\left(H_{T}-h_{m}\right)}^{2}}$ is the distance from the canopy edge to the receiver, (*λ*/4*πd*)^2^ and *T* are the same meaning as in section C. The derivation of Eq (14) has been explained in detail [28], the reasoning process is consistent with the electromagnetic wave attenuation model based on Tamir model, except that there is diffraction phenomenon of electromagnetic wave passing through vegetation canopy and Fresnel zone. If *x* is large, the ground reflections must be considered, because they partially cancel out the diffraction waves at the diffraction edge. Using Eq (12) and the first term of the Fresnel diffraction coefficient, we can further get Eq (14)’s third item as,

$$\frac{|D{|}^{2}}{\rho }=\frac{\lambda }{\rho {\left(2\pi \sin\theta \right)}^{2}}=\frac{\lambda }{\rho {\left(2\pi \theta \right)}^{2}}$$ (15)

Eq (15) is obtained according to the Fresnel zone geometric relationship of the wave incidence angle, when the electromagnetic wave passes through the vegetation canopy in Fig 3, we can calculate the path gain as follows:

$$PG=\frac{\lambda^{3}}{{\left(2\pi \right)}^{4}}\frac{1}{d^{4}}\frac{{\left(h_{BS}-H_{T}\right)}^{2}}{\rho \theta^{2}|\chi |}$$ (16)

### D. Wave propagation in rows of trees and buildings

**Fig 4:**
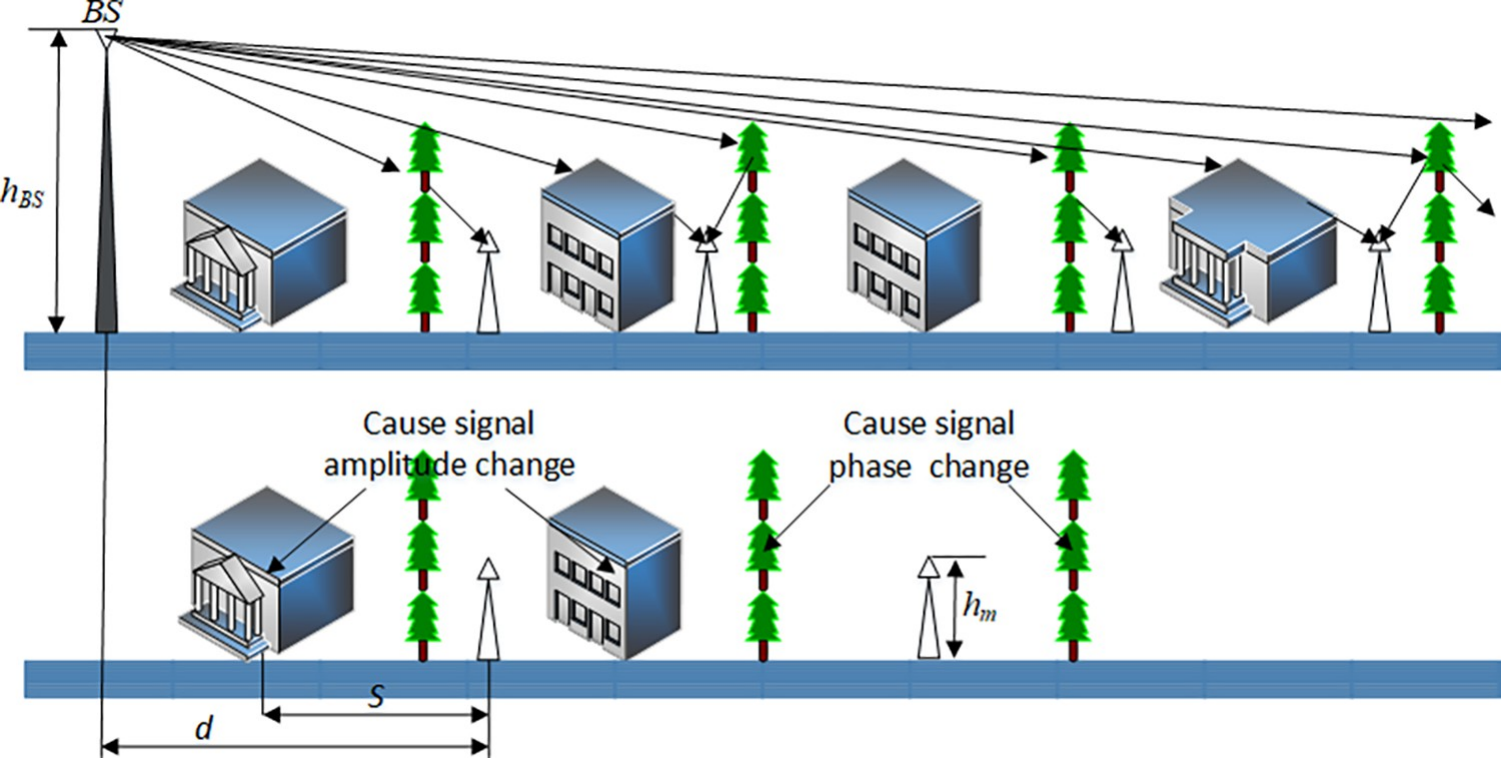
Electromagnetic wave propagation path with trees and buildings arranged in rows.

In urban and suburban areas, trees are usually arranged adjacent to buildings. Trees are usually two stories high, and their attenuation has a great impact on signal propagation. To simulate the effect of trees arranged in rows on propagation, only the ideal arrangement of trees and buildings is considered in the following analysis, as shown in Fig 4.

Microwave transmission is LOS. The building in Fig 4 can be regarded as an absorption wall that causes an amplitude change, the tree can be regarded as a partial attenuation wall, and the branches and leaves of the tree can cause additional phase changes. For simplicity, the attenuation wall and phase change wall of the trees in Fig 4 are superimposed directly on the absorption wall of the building.

Let the trees be described as a uniform triangle with their centre at the same height as the building. If the base length of the triangle is *a* and the long axis length is *b*, the tree width is:

$$w(z)=\frac{H_{T}-z}{b}\cdot a$$ (17)

**Fig 5:**
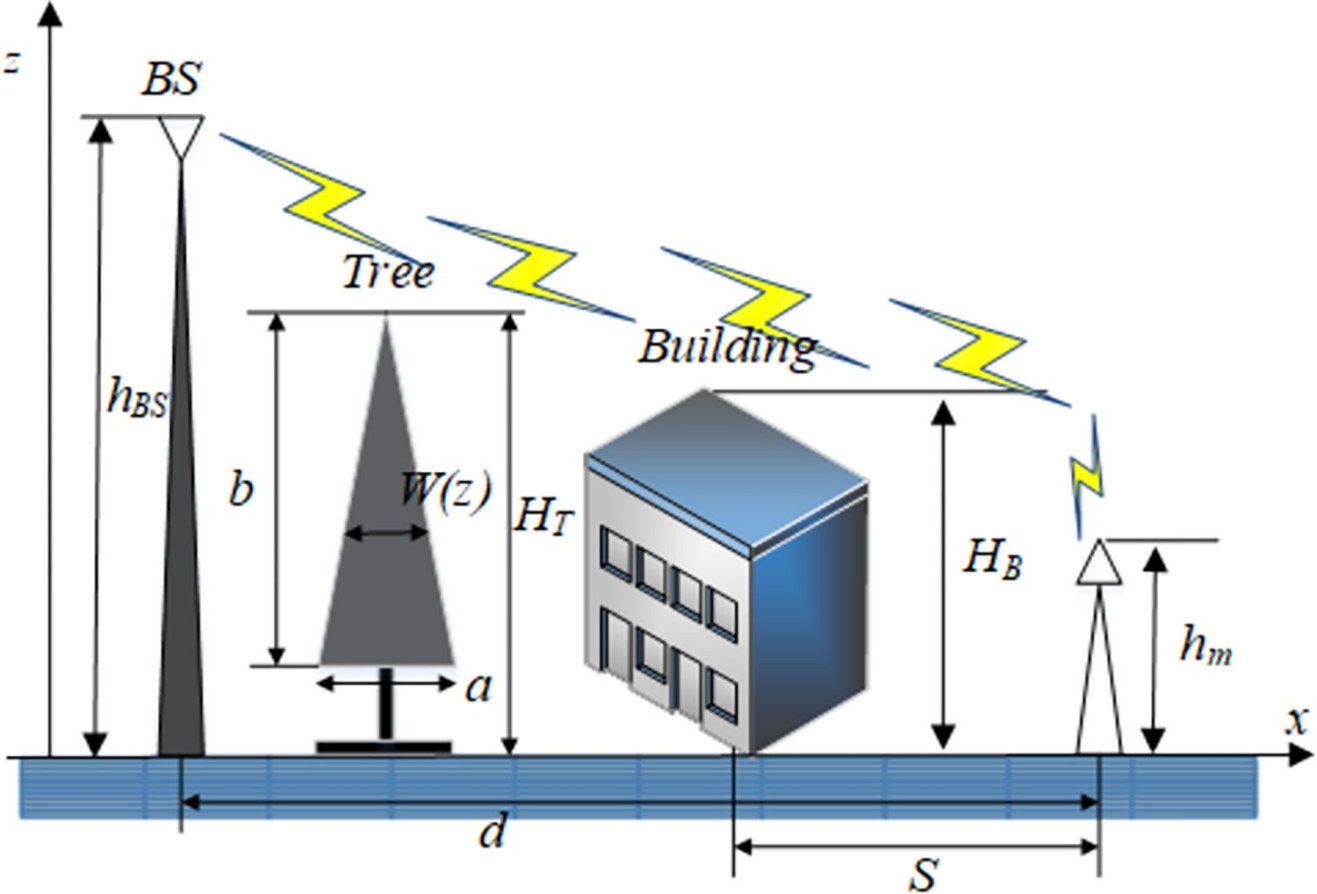
Diagram of triangular trees and buildings.

In combination with the analysis in sections D~E, i.e., according to Eqs (13) and (16), when *x* is larger, *ρ* = *ρ*_*G*_ and Γ_*G*_≈−1. According to the first term of the Fresnel diffraction coefficient, the gain of radio waves propagating in line with the trees and buildings can be obtained as follows (as shown in Fig 5):

PG=λ3(2π)41d4ρ|χ|(hBS−HT)2(HT−hm)2exp[2(HB−hm)⋅c⋅Im(χ)] (18)

Eq (18) is the formula for radio wave propagation path gain derived in this article. $\frac{\lambda^{3}}{{\left(2\pi \right)}^{4}}\frac{1}{d^{4}}\frac{\rho }{\left|\chi \right|}\frac{{\left(h_{BS}-H_{T}\right)}^{2}}{{\left(H_{T}-h_{m}\right)}^{2}}$ is the variation of signal amplitude derived from the above sections, exp[2(HB−hm)⋅c⋅Im(χ)] is the change of signal phase, and its derivation and principle are described in Section A. To verify the validity of this conclusion, assuming that the height of the base station is 30 m, the height of the tree canopy is still obtained according to [29,30]. When *x* = 50 m the basic path gain of electromagnetic waves can be obtained from Eq (18).

## Measurement scheme and system

### A. Channel large-scale fading characteristic measurement

We focus on the measurement of large-scale fading characteristics of wireless channel. There are two basic conditions for channel measurement of large-scale fading: first, the power of the measured signal is constant; second, the receiver can count and measure the channel parameters of the target signal. The data processing can be measured in the time domain, or obtained in the frequency domain through the spectrum analyzer.

By analyzing the characteristics of wireless broadband channel in the above chapter, this paper designs a set of channel test scheme suitable for scenario requirements according to the characteristics of complex channel environment, including continuous wave measurement method [27] and broadband signal measurement method [31].

### B. System measurement principle and method

**Fig 6:**
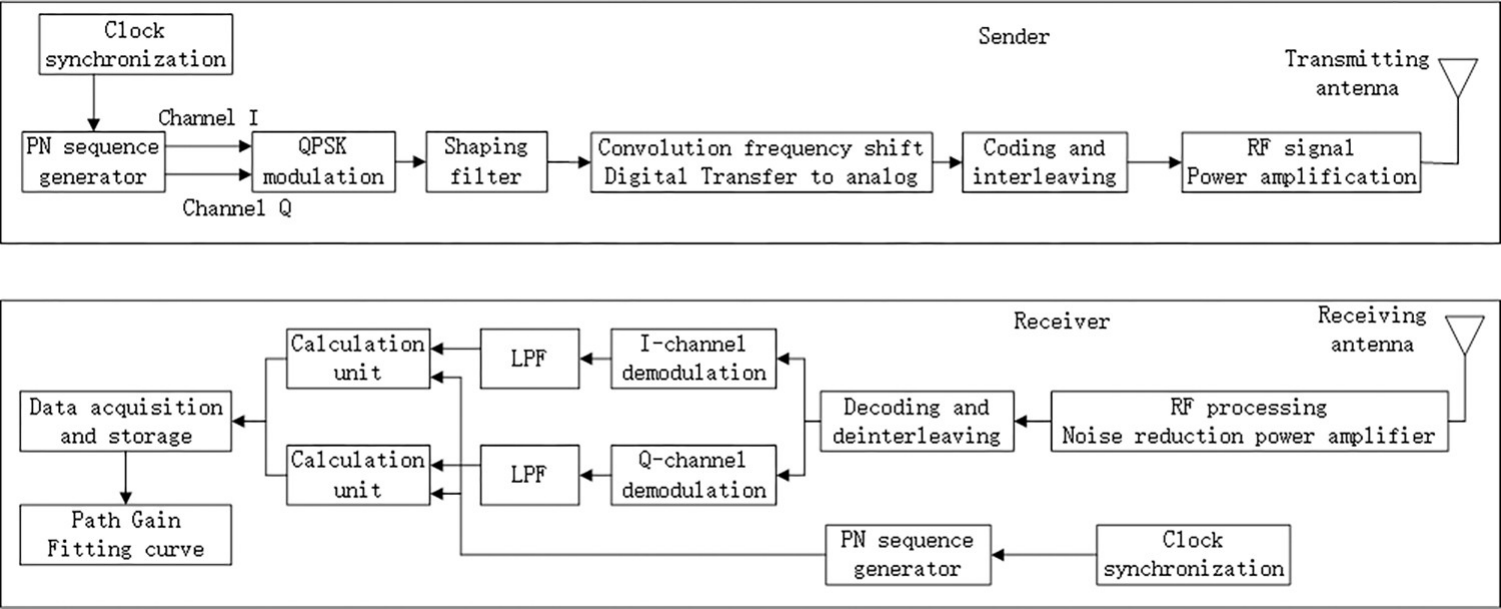
Measurement principle and method of self-built system.

When building the channel measurement system, the requirements and the equipment performance are considered. Because the spread spectrum communication has strong anti-interference ability and high measurement accuracy, this paper improves the spread spectrum communication, changes the packet capturing method of the original test system, captures the channel sampling information of the in-phase branch and orthogonal branch of the test signal. The sampling signal of the receiving is correlated with the local in-phase branch to obtain the real part, the sampling information of the quadrature branch is correlated with the local quadrature branch, to obtain the imaginary part. In this way, the amplitude and phase of channel impulse response can be obtained at the same time to complete the channel impulse response. The system principle is shown in Fig 6.

The measurement system is used to model the PG mentioned in the above chapter. According to the scenarios shown in Figs 1–4, the transmitter and receiver are placed in the relevant areas covered by vegetation. At the transmitter, the PN sequence generator contains the power, frequency, phase, amplitude and other parameter information of the original electromagnetic wave. The modulated signal is formed through shaping filtering and digital to analog conversion, the convolution frequency is moved to the measurement frequency point (200MHz~2.6GHz), the reason for choosing this frequency range is that this band covers all frequency ranges from 2G-5G, which consistent with the existing mobile communication cell frequency band. It is transmitted through RF antenna in the form of signal, and sent to the receiver through vegetation covered channel. At the receiver, the signal is demodulated and restored to the original electromagnetic wave. The output of the Path Gain Fitting curve unit is the attenuated fitting waveform, that is, the PG expression derived from Eqs (6), (13), (16) and (18). The PG model is determined according to the output of the unit.

After the measurement of above system, the receiver can obtain multiple maximum correlation outputs, then according to the distance between these maximum correlation output peaks, the multipath relative delay of signal can be calculated, in addition, the intensity of the signal passing through each path can also be calculated, without considering the conditions of multiple antennas, they can be calculated from the envelope of the in-phase and quadrature branch:

$$E(t)=\sqrt{I{\left(t\right)}^{2}+Q{\left(t\right)}^{2}}$$ (19)

### C. System parameters and equipment connection

For the signal extraction of the above measurement system, the peak value of the received signal can be obtained from the correlation between transmitting and local sequence at the receiver. The parameters to be measured:

1. Channel bandwidth: in order to meet the maximum channel bandwidth demand of the existing test system (LTE), all measurement scenarios adopt 20MHz bandwidth sequence.
2. Multipath identification: the multipath identification of the system determines the measurement accuracy. It is determined by the chip width of the transmission sequence. According to the Nyquist principle, when the delay interval of the channel is greater than twice the chip width, the multipath effect can be identified, that is:
$$\Delta \tau =2T_{c}$$ (20)
3. Maximum channel delay: according to the vegetation scenario, the carrier wave of the signal is 200MHz~2.6GHz. The measured delay that can be measured is greater than the maximum delay of the actual channel, but less than the channel coherence time, which is 418.35us, therefore, the delay of the channel impulse response is less than 418.35us. The design period is a time series of 1023. The period of the chip is 0.05us, the calculated sequence period is 51.15us, meet the requirements of maximum channel delay.
4. Coverage: as mentioned before, the coverage radius of the RRU is 1.5~5km, so during measurement, the signal power sent by the transmitter can cover the area, vegetation and buildings with a diameter of 3.0~10 KM, the power considers various losses on the path. The value of this coverage range is obtained by measuring the field strength, which is useful for sampling measurement and data processing of signal field strength in communication coverage area. The field strength tester uses a vertically polarized antenna. When measuring, the field strength tester is moved to record the measuring position and driving mileage, so as to sample when processing the measurement data. The data can be processed in real time or brought back to the data center for processing, the effective range of actual field strength coverage (i.e. the actual coverage area) can be obtained.

**Fig 7:**
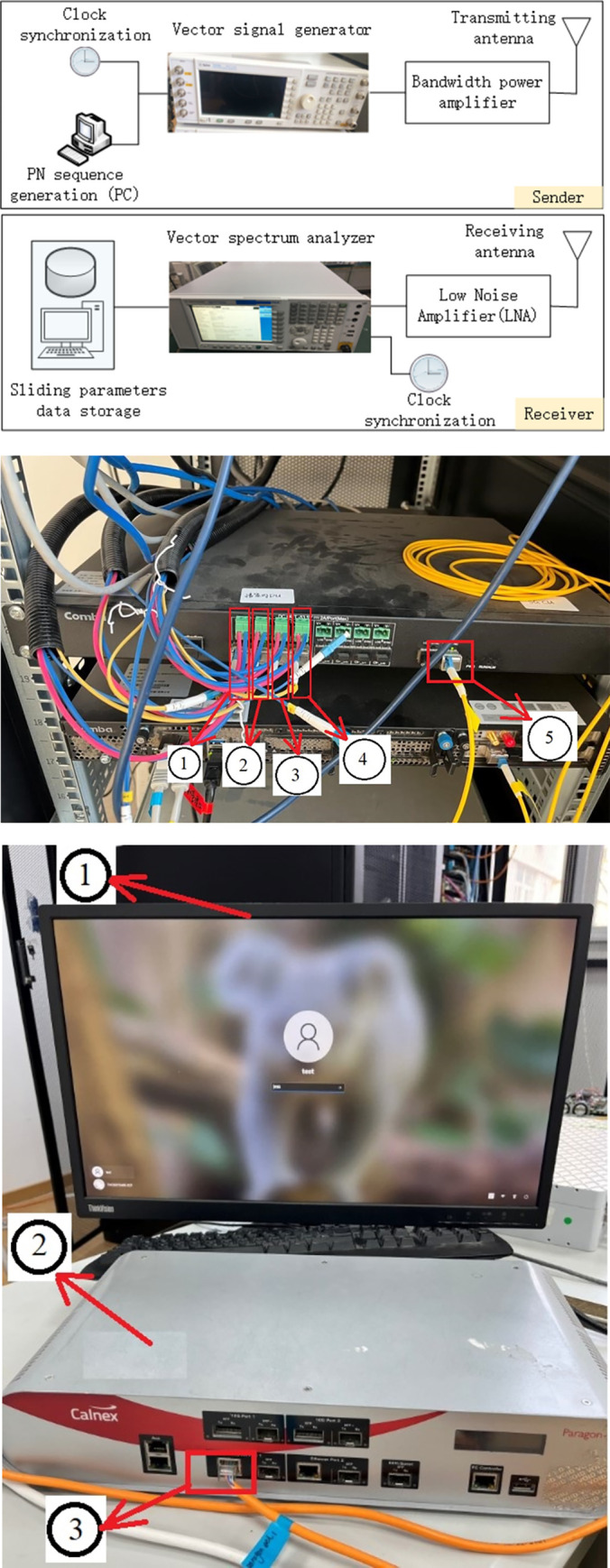
Self-built wireless channel measurement unit.

According to the requirements, schemes, measurement principles and parameters, a set of measurement system is built as shown in Fig 7. The content of this experiment is to obtain the relationship between channel parameters, large-scale fading and path attenuation, so as to find the channel model in vegetation environment and select the channel parameters which conform the actual transmission.

①Sender and receiver module. The RF transmission module is adopted, the sensitivity is -140 dBm, the maximum output power is 20 dBm, the receiving current is as low as 13 mA, sleep current is less than 10 uA, and the transmission rate is 0.018~37.5 Kbps. Within communication coverage, RSSI (Received Signal Strength Indicator) of receiver module is between (-50~-65) dBm.
②Test antenna. The test antenna is a rod antenna, the impedance is 50 Ω, the frequency is 200MHz~2.6GHz, the standing wave ratio is less than 2.0, and the antenna gain is 3 dbi.

The sender equipment is a signal generator manufactured by China Jingxin Network System Co., Ltd., the product model is ENB-5110E. The receiver equipment is a synchronous network analyzer and time-frequency integrated measuring instrument, which manufactured by Calnex Co., Ltd., the product model is Paragon-x. The site physical picture is shown in Fig 7(B) and 7(C).

### D. Performance simulation and verification of self-built system

According to the large-scale fading and multipath effect of scene channel, the wireless channel model parameters (single input single output) are constructed through the rayleighchan (sampletime, maxdoppler shif, delayvector, gainvector) function in the experiment as follows:

**Table:** 

sampleTime = 1/5000000; % sample time maxDopplerShif = 1; %Max Doppler Shif delayVector = 1.0e-0.005*[0.02 0.04 0.06]; %Discrete delays of Vector three-path channels gainVector = [–3 –6 –9]; %Average path gains (dB)

**Fig 8:**
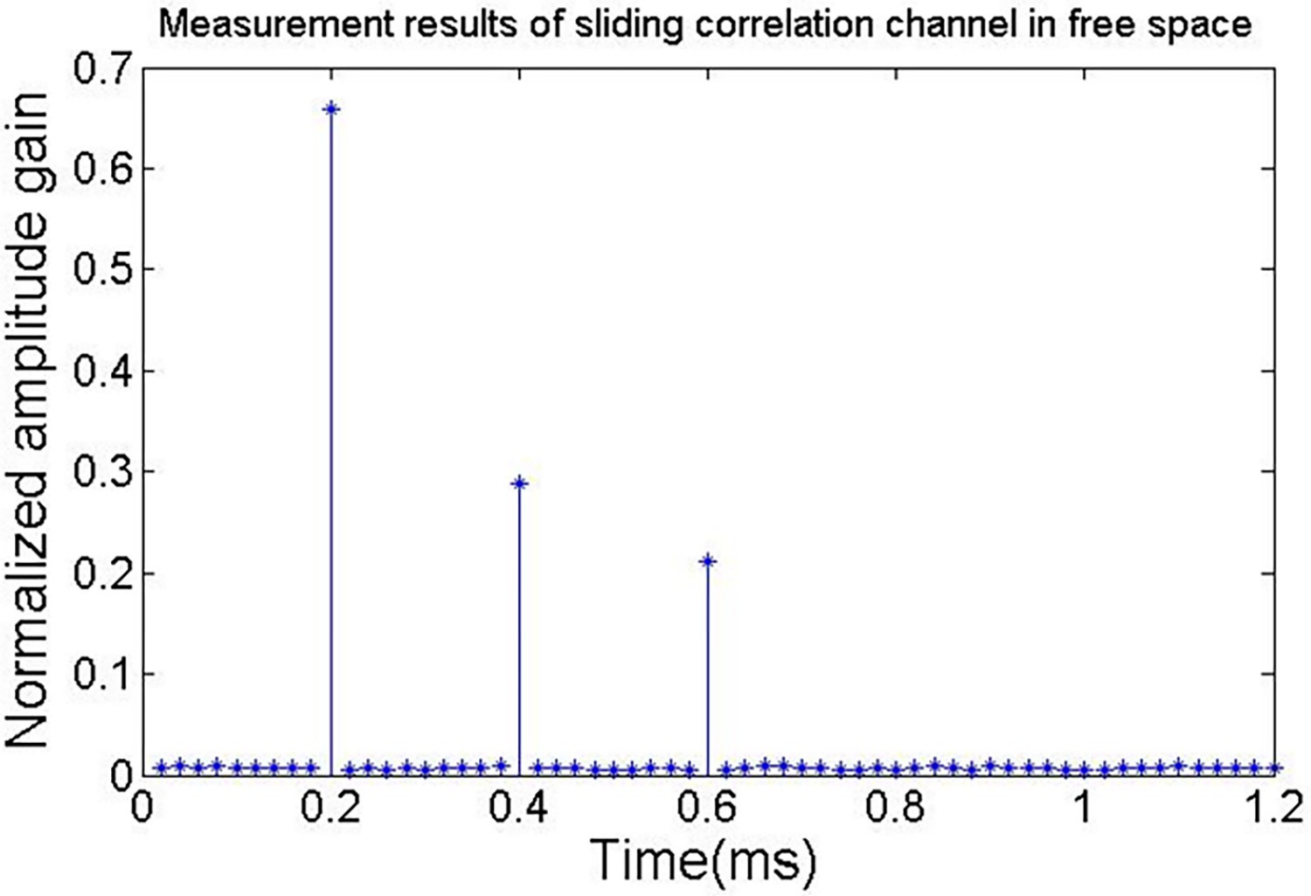
Channel impulse response.

Three multipath wireless channels are established, the average power gain of each path is -3 dB, -6 dB, -9 dB. the delay of each path is 0.2ms, 0.4ms and 0.6ms, at both end of the signal, the in-phase and orthogonal branch are sampled and captured to obtain the real and imaginary parts of channel impulse response, the I-channel sampling information corresponding to the real part, the Q-channel sampling information corresponding to the imaginary part. According to the above settings, the experiment result of the channel impulse response is shown in Fig 8.

It can be seen from Table 1 that the theoretical calculated value of channel multipath gain is consistent with the measured value, which indicates that the measurement system is accurate. According to the results of simulation, the scale fading and delay of the channel can be determined by using spread spectrum communication. At the same time, the multipath attenuation of the channel can be extracted from the sliding results by setting appropriate noise, multipath number, decision threshold and other parameters.

**Table 1:** Capture channel parameters of I and Q channels.

Delay(ms) Amplitude gain	Path 1 (0.2)	Path 2 (0.4)	Path 3 (0.6)
Impulse response gain	0.66183	0.29824	0.21581
Measure amplitude gain	0.65913	0.28741	0.21087

## Model experiment in scenario and analysis

**Fig 9:**
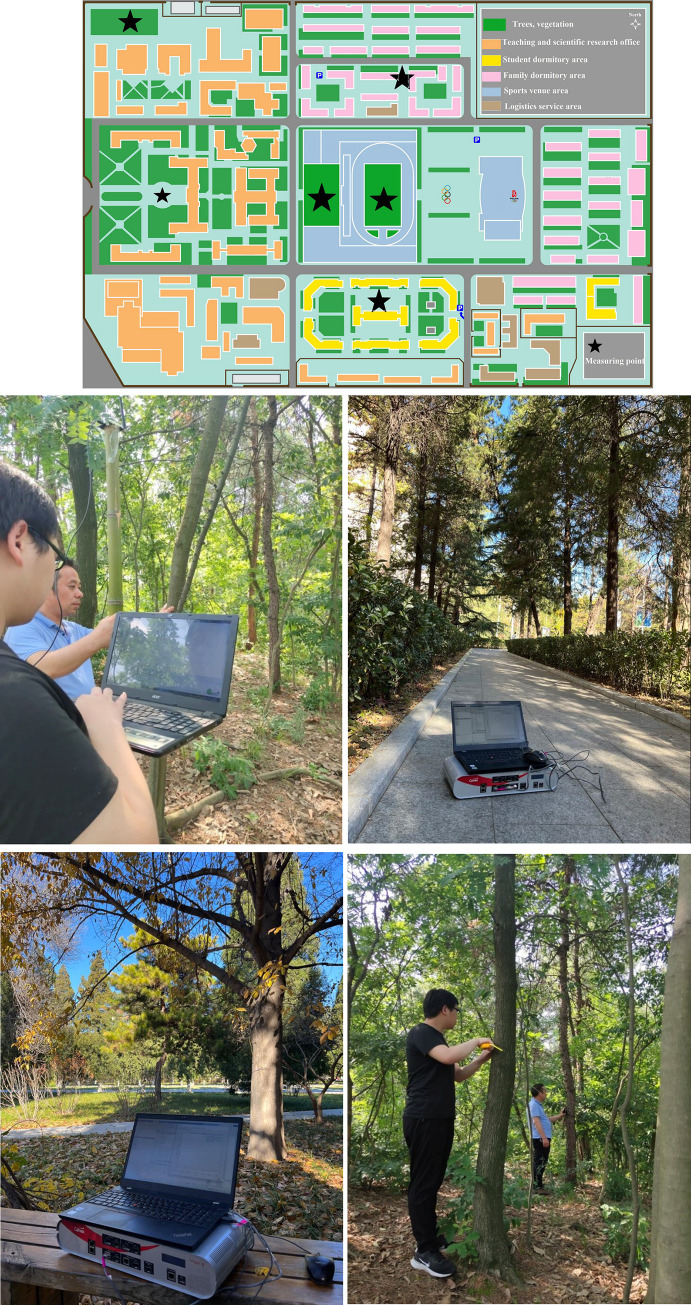
Channel measurement point map of Beijing University of Science and Technology.

After the numerical calculation and simulation verification, the channel measurement system (Fig 7) constructed can be used for large-scale channel fading test in vegetation environment. Therefore, we conducted multiple measurements in the map (Fig 9), selected 200MHz~2.6GHz without interference as the measurement frequency.

As shown in the green block in Fig 9, there are plant coverage scenes all over Beijing University of Science and Technology, covering an area more than 6 hectares, with trees, shrubs and grasslands, with different vegetation densities and heights of trees. The campus scene of the university is very suitable for channel measurement, meeting the requirements of the four scenarios mentioned in the article. The measurement locations are set at six locations of the pentagram in Fig 9, which are vegetation coverage areas, green square area, playground area, student and faculty dormitory building area correspond to the four scenarios described in above Chapter.

During the measurement, the spectrometer moves at a constant speed on the receiving side, receives the signals from the transmitting in real time, calculates and stores the sampled signals, and performs post-processing. After wavelet noise reduction, filtering, data smoothing and other operations on the signals of channel I and Q, the path gain value of each measurement point is obtained, the path gain value *PG* is obtained by fitting at the reference distance *D*. The measurement results of each group scenes are given below.

### A. Located in the vegetation covered area

**Fig 10:**
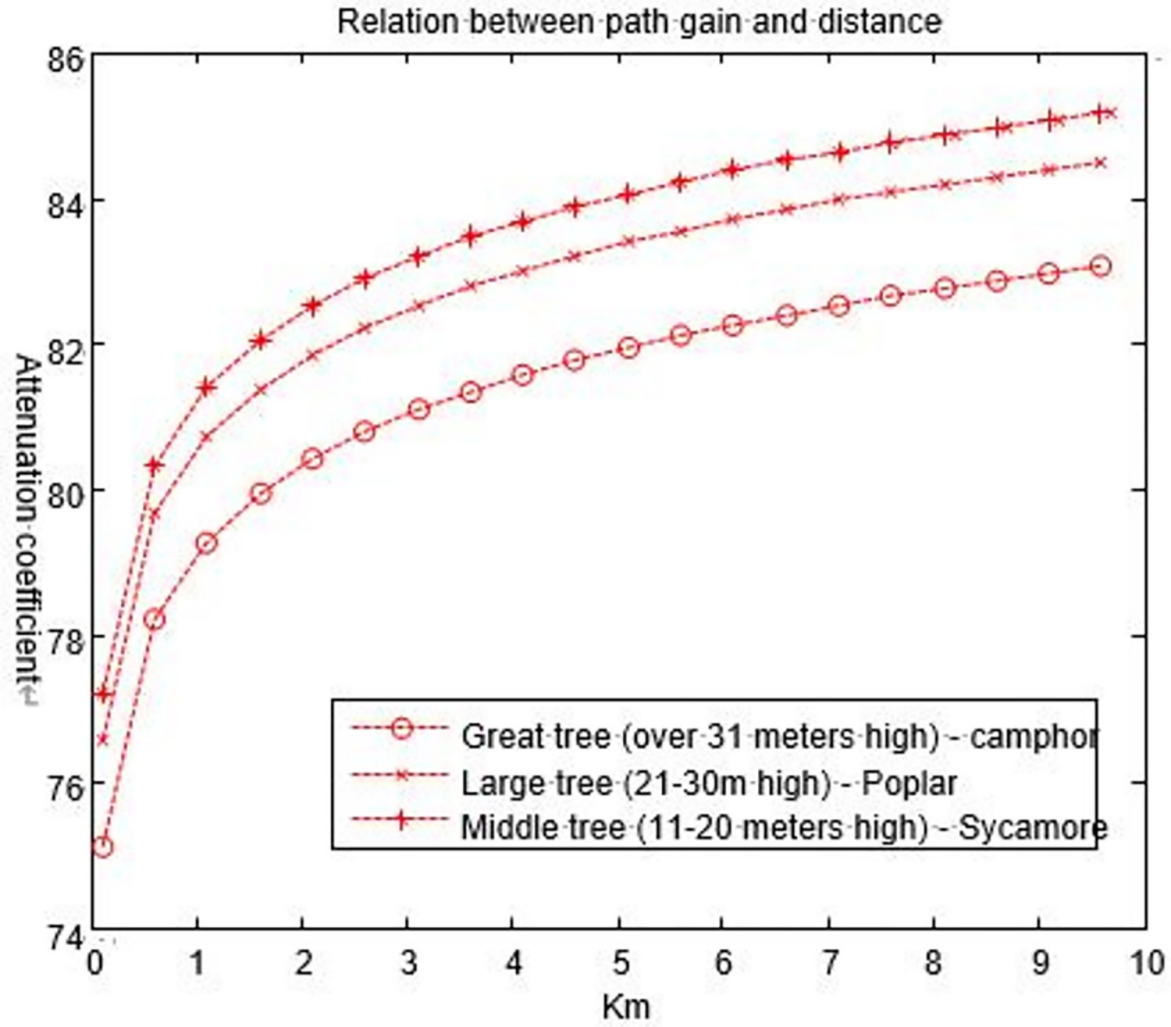
Relationship between the path gain of electromagnetic waves and the height of vegetation (the base station is located inside the vegetation).

Measuring range (*S*) is the sum of the distances from each moving end to the vegetation canopy, where the canopy height is *H*_*T*_. The propagation path is supposedly symmetric, i.e., *h*_0_ = *h*_1_. According to the vegetation statistical results in [29,30], the different heights of trees must be considered.We make the height of a base station be 5 m. The great trees, large trees and middle trees that have been growing for many years are taller than the base station; however, the small trees and shrubs are shorter than the base station, so we do not consider them here. Eq (6) is used to determine the basic path gain of electromagnetic waves in the process of vegetation propagation. The numerical calculations are shown in Fig 10.

According to the results in Fig 10, in a large vegetation, the path gain of an electromagnetic wave signal shows a logarithmic growth trend with an average attenuation of approximately 81.3 dB. The path gain increases with increasing distance of electromagnetic wave propagation in the vegetation, but the increasing range decreases with the increase in distance. When the propagation distance is less than 1 km, the slope of the electromagnetic wave gain curve is very large, and the signal attenuation is severe. When the propagation distance is more than 2 km, the basic path gain decreases with the slope of the propagation distance, and the speed of the signal attenuation slows down. In addition, compared with the three curves, the top curve is the middle tree. That is, the looser the tree structure is, the denser the plant leaves; the larger the dielectric constant is, the stronger the signal attenuation.

### B. Vegetation path in free space

**Fig 11:**
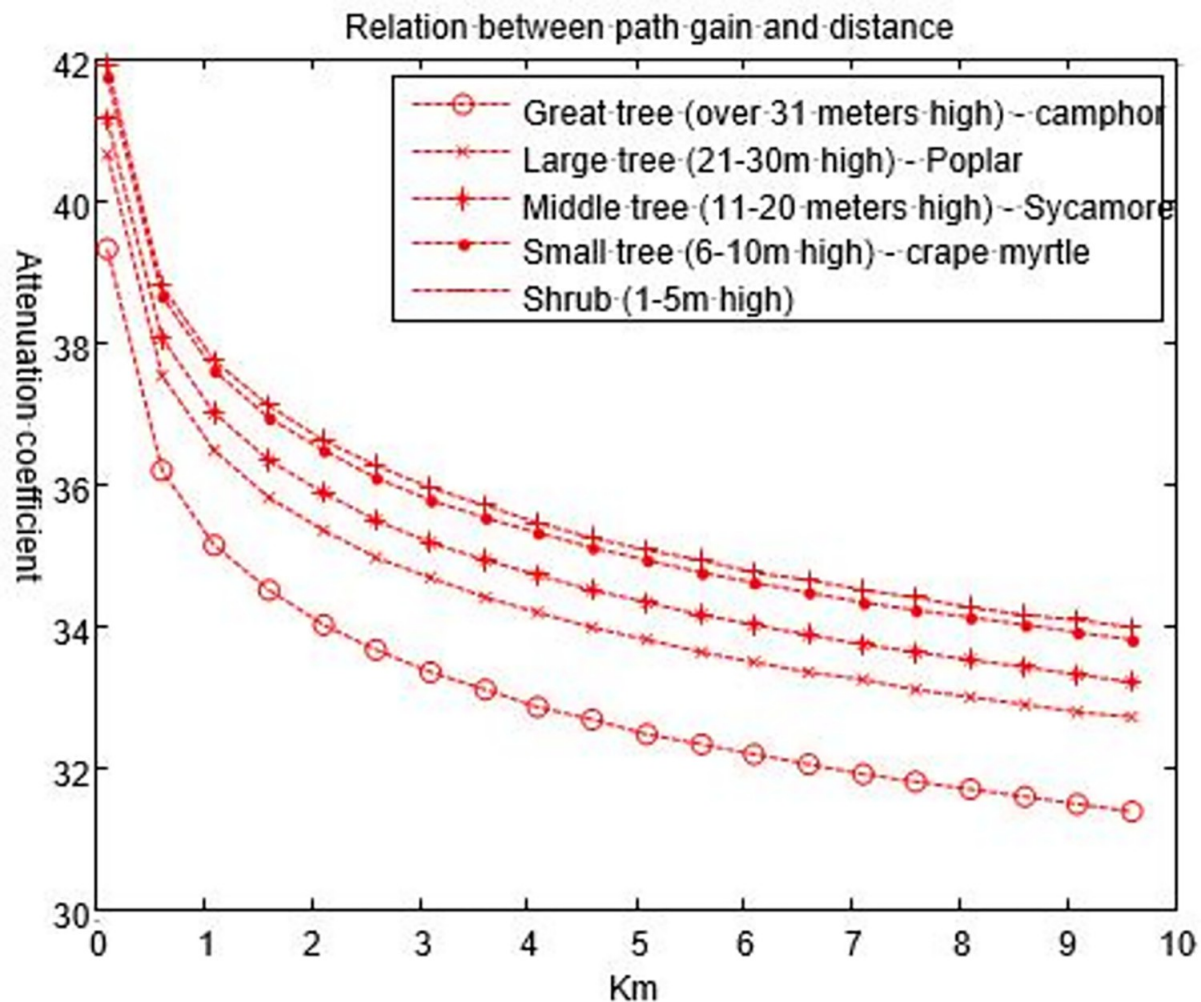
Relationship between the electromagnetic wave path gain and the distance with different vegetations in the free space of the base station.

In the communication from a mobile station to receiver, the received signal has a range factor 1/*d*^4^. According to the assumptions in the previous section, the height of a transmitter set in this section is 30 m, and the height of the canopy is still obtained according to [29,30]. Here, the shrubs and small trees are also included. The receiving antenna is set in the vegetation, and the height is half of the canopy height. The parameters have the same settings as those in the previous section: Use Eq (13) to calculate the basic path gain of electromagnetic waves during the propagation of vegetation, as shown in Fig 11. The average path gain in 200 MHz is approximately 36.5 dB, of which 9.5 dB is caused by trees at the base station.

It can be seen from Fig 11 that under different types of plant cover, the path gain of electromagnetic waves decrease with increasing propagation distance, and the attenuation amplitude decreases with increasing distance, which is completely opposite of the conclusions in the previous section. However, at the same distance within 1 km, the signal slope is large and the attenuation is serious because the scattering, diffraction, absorption and other phenomena are frequent when the signal is transmitted over a short distance. These physical phenomena are the main parameters that affect the signal quality. With increasing distance, the distance of the signal transmission in free space is longer and the phenomenon of electromagnetic wave absorption, reflection and cancellation by plants is gradually weakened. The signal transmission is only affected in the canopy layer. According to Eq (13), the farther the distance is, the closer the relationship with *d* is, and the weaker the correlation with the density, water content and other factors of the vegetation itself. In addition, it can be seen from Fig 11 that the shrub has the greatest attenuation, which also shows that the water content, density, dielectric constant, etc. of the plant are still important factors affecting the signal transmission. From the comparison of results in Figs 10 and 11, the path gain from the mobile station to the mobile station is 44.8 dB greater than that from the base station to the mobile station. Although Eq (6) is still applicable when the base station antenna in the vegetation rises above the canopy, Eq (13) is applicable only when the base station is several wavelengths higher than the canopy.

### C. Signal propagation in open space of vegetation

**Fig 12:**
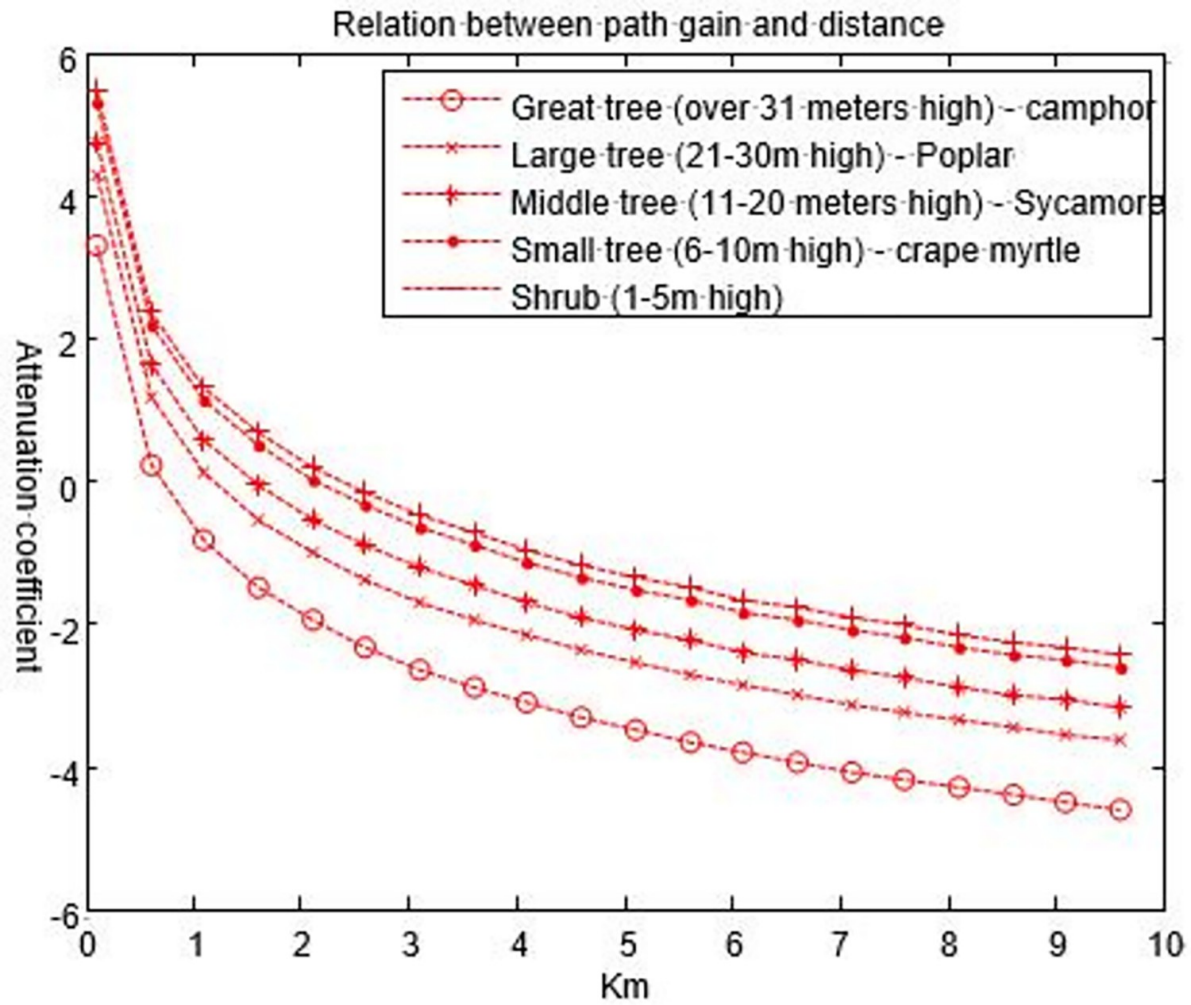
Relationship between the path gain of electromagnetic waves through different vegetations and distance.

The distance factor (1/*d*^4^) existed in the previous vegetation propagation model. To compare the different path gains of section 2’C and D, we assume the height of the base station is 30 m, the height of tree canopy is still obtained according to [29,30],. The basic path gain of the electromagnetic wave can be obtained from Eq (16), as shown in Fig 12.

It can be seen from Fig 12 that the electromagnetic wave signal is mainly transmitted in free space, the role of vegetation is equivalent to diffraction, and the value of signal attenuation is greatly reduced. In the 200 MHz frequency band, the average path gain is approximately 1 dB, which is approximately 80 dB less than the path gain in section 2’C, and 35 dB less than the path gain in section 2’D. This finding shows that the diffraction gain is greater than the attenuation in the canopy under the above parameters. In the higher frequency band, the attenuation caused by the tree canopy will make the attenuation in the vegetation greater than that in hollow ground.

### D. Trees and buildings line up

**Fig 13:**
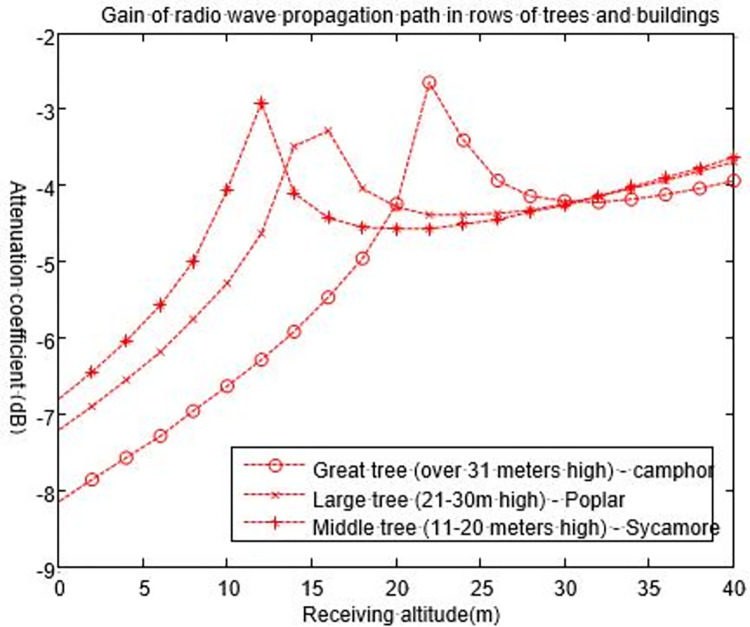
Path gain using the triangle tree model.

Fig 13 shows the result when the incident frequency is 200 MHz for a plane wave obtained by Eq (18), assuming that the distance between the base station and the mobile station is *d* = 50 m, the height of the double-layer building is *H*_*T*_ = 12 m (*b* = 8 m), and the maximum width of trees is 4 m (*a* = 4 m).

Fig 13 shows the path gain of a wave to the 20th row of trees and buildings when the distance *S* = 20 m and the incident plane wave angle is *α* = 0.5°. Although the width of trees is different, their influence on the field strength is similar to that of buildings without trees. After removing the maximum depth interference caused by other multipaths, the signal of users on a street is 5 dB lower than a street without trees.

It can also be seen from Fig 13 that when the height of a receiving station is less than 20 m, the signal attenuation and gain are greatly affected by great trees. This influence is observed is because great trees are relatively tall. Generally, great trees growing for several years can reach heights greater than 30 m, which means that the base station is located below the tree. At this time, the transmission of the signal through the plant canopy will inevitably cause great attenuation. When the height of the base station is between 20–30 m, the height of the receiving station is further increased; the height of the receiving antenna is higher than that of the three types of vegetation, and the attenuation of the signal is mainly caused by free space and the shelter of buildings. In addition, it can be seen from the figure that the influence of the three vegetation types on the signal decreases with the increasing receiving height of the mobile station. After exceeding a certain height (20 metres), the signal attenuation increases, and then after a certain height (30 metres), the signal attenuation gradually decreases.

**Fig 14:**
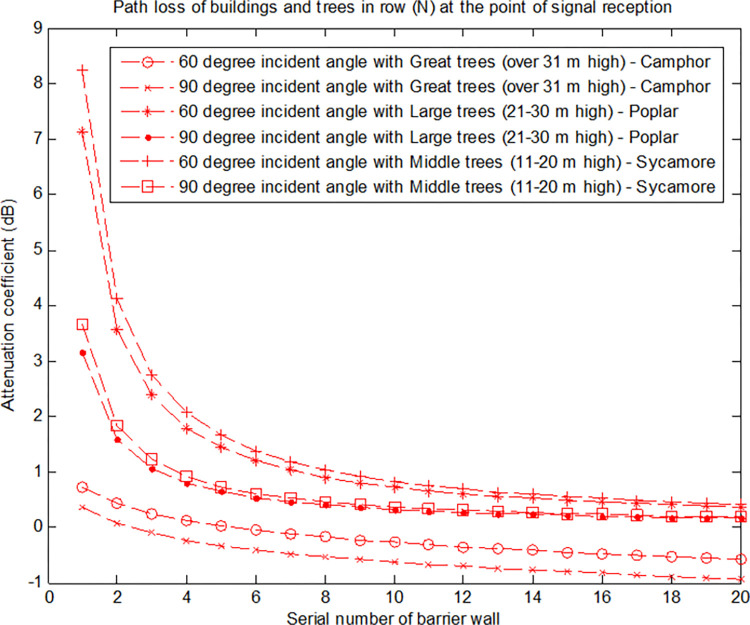
Path gain of buildings and trees in row N at a signal receiving station (varying with angle).

Similarly, according to the above setting parameters, we analyse the signal attenuation of the base station incident to the mobile station target at different angles, as shown in Fig 14.

Fig 14 is the path gain obtained by the N-th row of buildings at the signal receiving station (*H*_*B*_) when the incident angles of plane waves are 90° and 60°. The results show that losses tend to be constant when the incident angle is 60°, and the field strength decreases with N when the incident angle is 90°. However, when N increases, the difference between the two curves with and without trees will be closer to a constant value between 1 dB and 2 dB. As the influence of branches is only half of that caused by both branches and leaves, the path gain will be reduced by 2~3 dB when leaves fall off in winter.

### E. Large scale fading in different scenarios

From the above scenarios, we analyze the changes of large-scale fading parameters. By shadow fading factor:

S=PL(d)−PG−10n⋅log(d) (21)

Where *n* is the path gain index. Eq (21) is the shadow fading factor, which is the difference between the estimated and measured value of path gain. The comparison and analysis of large-scale fading parameters under the four scenarios are summarized in Table 2.

**Table 2:** Channel large-scale fading measurement statistics (points in Beijing University of science and Technology).

Location	Path gain (PG)	Path gain index (n)	Shadow fading standard deviation (*σ*)
Located in the vegetation covered area	81.3	2.8	7.1
Vegetation path in free space	36.5	2.4	4.8
Signal propagation in open space of vegetation	1.6	3.2	10.1
Trees and buildings line up	1.5	3.0	9.2

It can be seen from Table 2, when both the transmitter and receiver are located in vegetation covered area, the signal path gain is increased with the increasing of distance, in the other two cases, the signal has a free space propagation process, only when it enters the vegetation, refraction, diffraction, scattering and other effects occur, so the signal path gain is decreased with the increasing of distance. In addition, between the transmitter and receiver, due to the influence of environment factors such as vegetation density, tree height, building shelter, etc., the channel change will be affected, this can be verified through the change of *PG* by which caused the same propagation distance in different scenarios.

Through further analysis, it is known from experience that the large-scale path gain index in free space is 2. In the scene covered by vegetation or buildings, the variation range of this value is considered to be 2~3.5. The path gain index values of the measured points are within this range, which confirms the conclusion that the line of sight propagation path gain index value is greater than 2 when there are obstacles in space.

Finally, the influence of vegetation coverage can also be analyzed through the *σ*. The value of *σ* reflects the change of received power at the point caused by random factors such as reflection, absorption and scattering. As several group of scenes tested have direct path, the *σ* value of several group is not large, especially in the open sight distance environment, such as the dormitory area of students and teachers, the received power is stable, so the median fluctuation of signal power is small. The shadow declines in the green square area, due to the large vegetation coverage around, the signal transmission path does not penetrate through the plants themselves, resulting in unstable measurement power, the shadow fading of this scene has a great impact.

### F. Comparison of different channel fading models

**Fig 15:**
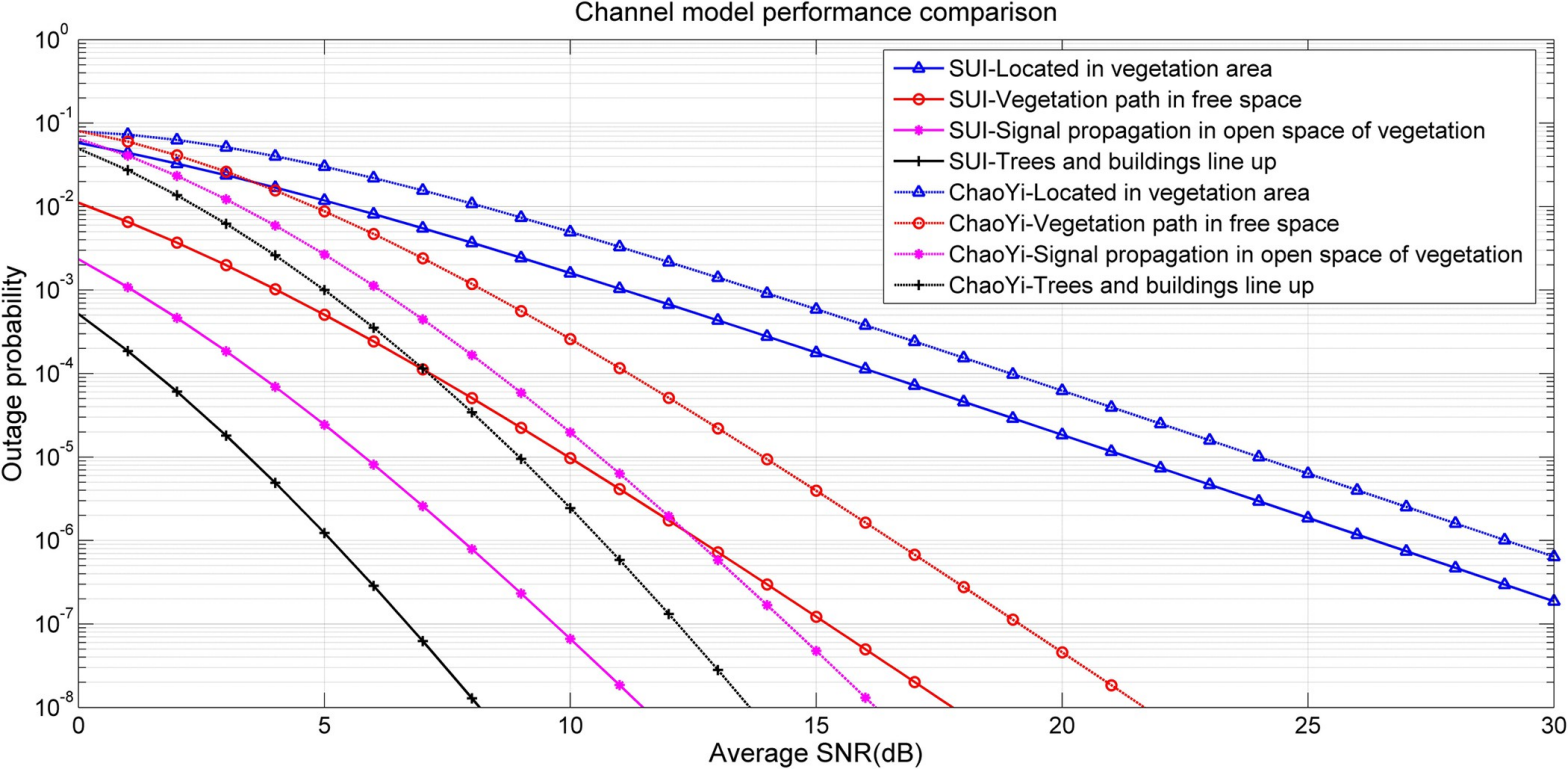
Channel model performance comparison.

For the fading parameter in different scenarios, the number of multipath and delay characteristic of channel need to be analyzed. In the previous experiment, the delay characteristic of different scenarios is extracted to obtain the channel’s power delay line model. Next, we compare and analyze two models, one is the SUI channel model proposed in [32], which can divide the suburban path gain into three terrain types according to the tree density and path gain. It is also the closest channel to the model proposed in this paper. The models under four scenarios proposed are called ChaoYi channel model in this paper. In this section, the 3-path taps of ChaoYi model and SUI model are fitted with the actual measured data, the results are compared and analyzed, as shown in Fig 15.

Fig 15 shows two model’s comparison of outage probability under different average SNR (i.e. different sample number) in the above four scenarios, they are with tapped delay lines and delay sampling points. SUI model and IEEE 802.16d channel have the same application, so it is applicable to the suburb’s three terrain type. The parameters (frequency, bandwidth, etc.) of the ChaoYi channel model is similar to the SUI model, however, it is an internal environment, there is no undulating terrain, slope and other scenario, so it is different from the environment defined in standard. In the ChaoYi channel model, the number of multipaths is set as a fixed value in general vegetation coverage scene. There is a gap between the fitting degree of simulation and measurement system.

Since the model is applicable to forests, campuses, parks and other environment with vegetation coverage, the measurement results show that the difference between the channel model and the general vegetation coverage environment is small, the difference between the channel model and the scenes with terrain, ocean or city is large. This also shows that when establishing the vegetation channel model, it is necessary to consider the impact of various environment factors (such as climate, terrain, etc.), and the spacing between receiver and transmitter. In comparison, the four environment tapped delay line models, which based on different scenario, are obtained according to the sampling statistical characteristics of the channel. This approach will better fit the channel characteristic of the vegetation coverage scene, through adjusting model parameter as the number of sample increasing, especially the physical phenomena such as absorption, diffraction and scattering by vegetation is the most obvious, this characteristic also affect the distribution of power delay at the receiver. Through the above analysis, the ChaoYi channel model proposed in this paper has a good fitting degree in vegetation coverage, the interruption probability of the sample is small.

## Conclusion

This paper researches four situations of the electromagnetic wave transmission under the condition of vegetation coverage, carries out theoretical derivation and numerical simulation for the signal attenuation of each case. The content is summarized as follows:

1. According to the experiment results, the PG without or less occlusion is higher than that in the environment with vegetation shelter, the gap is more obvious with the increasing of communication distance. The higher coincidence degree between antenna and tree leaf height, the smaller PG will be.
2. The results show that the PG of four scenarios are 81.3 dB, 36.5 dB, 1.6 dB, 1.5 dB, the value of path gain index is within the range of 2~3.5, four scenarios shadow fading standard deviation values are 7.1, 4.8, 10.1, 9.2, reflects the change of received power at the point caused by random factors such as reflection, absorption and scattering. In addition, the proposed channel model improves the gain about 15% compared with the tradition SUI model within vegetation coverage scene.
3. When applying this channel in vegetation environment, it is necessary to deploy the node location within the monitoring range according to the distance and environment requirement. At the same time, the tree height, antenna height, vegetation environment changes are also necessary to consider.
4. In future, we will study the impact of environment parameters such as weather and forest density on the channel, especially the change of vegetation dielectric constant, establish a relationship model of dielectric constant on channel modeling, build a wireless channel model more suitable for vegetation coverage environment, provide a theoretical basis for electromagnetic wave transmission in various frequency bands.

## Supporting information

S1 File Supplementary material. (RAR)
